# Chickpea seed endophyte *Enterobacter* sp. mediated yield and nutritional enrichment of chickpea for improving human and livestock health

**DOI:** 10.3389/fnut.2024.1387130

**Published:** 2024-04-25

**Authors:** Arpan Mukherjee, Anand Kumar Gaurav, Gowardhan Kumar Chouhan, Saurabh Singh, Ankita Sarkar, Saman Abeysinghe, Jay Prakash Verma

**Affiliations:** ^1^Plant Microbes Interaction Lab, Institute of Environment and Sustainable Development, Banaras Hindu University, Varanasi, Uttar Pradesh, India; ^2^Department of Mycology and Plant Pathology, Institute of Agricultural Science, Banaras Hindu University, Varanasi, Uttar Pradesh, India; ^3^Department of Botany, Faculty of Science, University of Ruhuna, Matara, Sri Lanka

**Keywords:** seed endophytes, nutrient, microbiome, chickpea (*Cicer arietinum* L.), *Enterobacter* sp.

## Abstract

Chickpeas (*Cicer arietinum* L.) are used as a good source of proteins and energy in the diets of various organisms including humans and animals. Chickpea straws can serve as an alternative option for forage for different ruminants. This research mainly focussed on screening the effects of adding beneficial chickpea seed endophytes on increasing the nutritional properties of the different edible parts of chickpea plants. Two efficient chickpea seed endophytes (*Enterobacter* sp. strain BHUJPCS-2 and BHUJPCS-8) were selected and applied to the chickpea seeds before sowing in the experiment conducted on clay pots. Chickpea seeds treated with both endophytes showed improved plant growth and biomass accumulation. Notably, improvements in the uptake of mineral nutrients were found in the foliage, pericarp, and seed of the chickpea plants. Additionally, nutritional properties such as total phenolics (0.47, 0.25, and 0.55 folds), total protein (0.04, 0.21, and 0.18 folds), carbohydrate content (0.31, 0.32, and 0.31 folds), and total flavonoid content (0.45, 027, and 0.8 folds) were increased in different parts (foliage, pericarp, and seed) of the chickpea plants compared to the control plants. The seed endophyte-treated plants showed a significant increase in mineral accumulation and improvement in nutrition in the different edible parts of chickpea plants. The results showed that the seed endophyte-mediated increase in dietary and nutrient value of the different parts (pericarp, foliage, and seeds) of chickpea are consumed by humans, whereas the other parts (pericarp and foliage) are used as alternative options for forage and chaff in livestock diets and may have direct effects on their nutritional conditions.

## 1 Introduction

World populations rely on obtaining the necessary amounts of essential micronutrients from their diets to support normal physiological functions and maintain health. Billions of people around the world do not receive sufficient amounts of several important micronutrients due to low concentrations of available nutrients present in the grain/staple/seeds of food crops. In developing countries, most children under the age of 5 suffer from nutrient deficiency, which causes major health issues (1–3). Although the most conventional and fruitful strategies were mineral supplementation with food, dietary food diversification, and food fortification, the idea was not well spread due to a lack of social awareness and economic infrastructure (4, 5). To maintain the basic need for food supply, the production of legume seeds requires a very significant jump over time. In order to maintain the basic food supply and achieve such a huge target, current agriculture mostly depends on chemicals such as pesticides and fertilisers (6, 7). The application of chemicals as fertiliser directly and indirectly causes adverse effects on the environment and human health (8). Therefore, priority should be given to the biofertiliser (green manuring, crop rotation, vermicompost, etc.). Amongst all the alternative options, the application of plant growth-promoting microbes (PGPMs) has been identified as the best option and least explored area of research that can be applied to improve agriculture production without affecting the environment or human health (9–11). The application of biofertilisers in the agriculture sector has been observed to improve mechanisms, grain host plant growth, health and defence mechanisms, grains yield, and nutrient content, as well as the signalling pathways related to stresses (12). Recently, organic fertiliser inputs have been recommended as one of the safe alternative options for maintaining soil health and thereby enhancing the organic nutrient management of agriculture fields (13).

Chickpeas are one of the most important legume grains, and they are used around the world as a rich source of nutrients (vitamins, minerals, proteins, carbohydrates, etc.) for daily use in diets (14). Usually, chickpea leaves are used as leafy vegetables to supply huge amounts of nutrients/minerals/proteins/vitamins to people in many countries. Chickpea pericarp and straw are the main by-products of the chickpea plant and also serve as high-nutrient dietary fodder for livestock (15). From the seed to the straw, all parts of chickpeas are consumed as a high-nutrient source for humans and livestock. Moreover, each part of chickpea plants is consumed by different animals at different stages.

**Figure 1:**
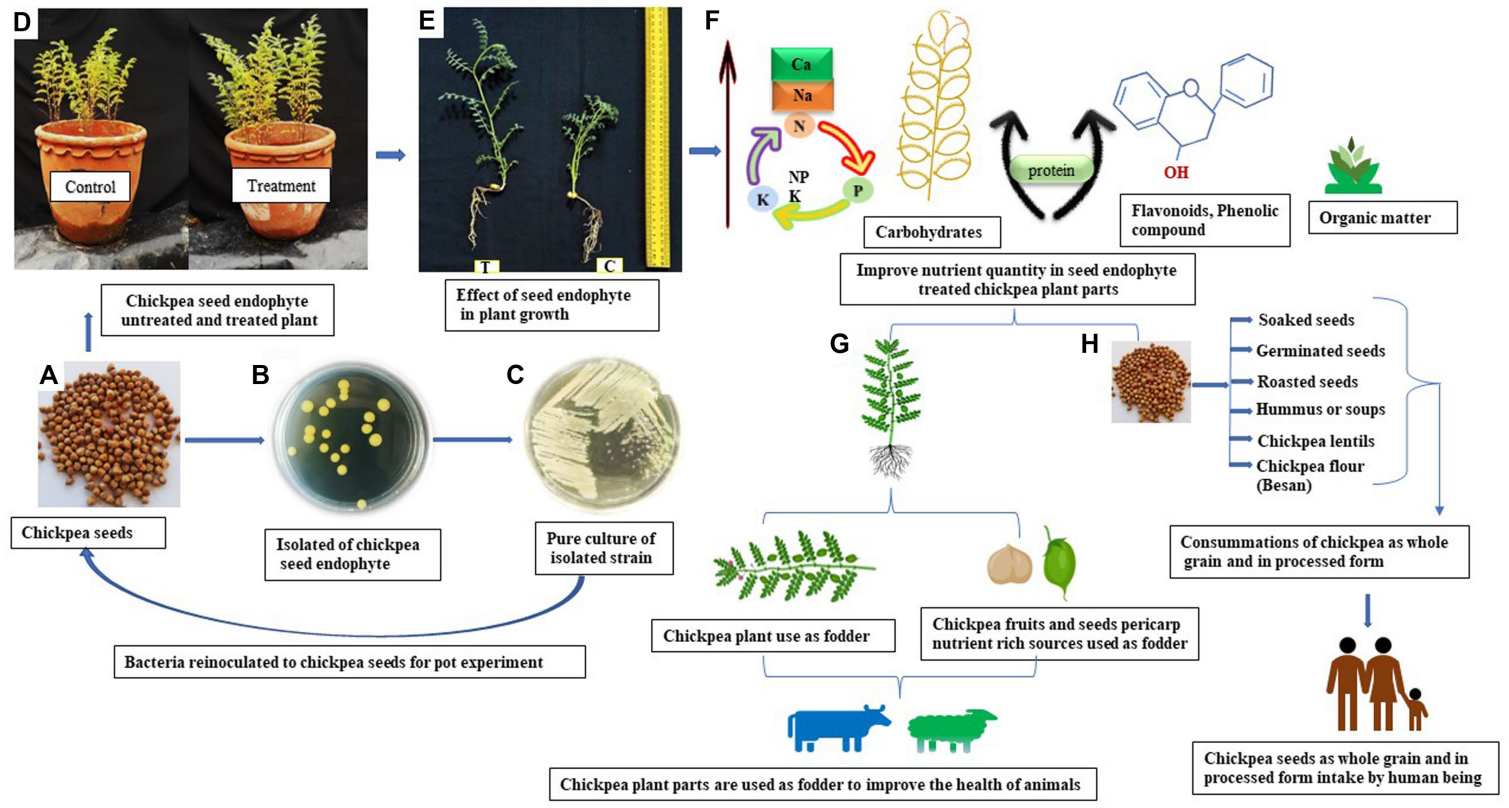
Diagrammatic presentation of the application of the chickpea seed microbe in plant growth and nutrient management in chickpeas. **(A)** Chickpea seed (P-362), **(B)** isolated chickpea seed endophytes, **(C)** pure culture of the isolated strains, **(D)** re-inoculated chickpea seed endophytes in chickpea seed for pot trials, **(E)** effect of endophytes on the plant growth and development (C **=** control plant and T **=** Seed endophyte-treated plants), **(F)** improvement of the nutrient in the seed endophyte-treated plant parts (seed, foliage, and pericarp), **(G,H)** Nutrient-rich chickpea (seed, foliage, and pericarp) used for human consumption as well as fodder for improving health.

PGPMs have been successfully used either as biofertilisers or as bio-pesticides for a very long time (16). However, the application of the seed endophytes to plant growth promotion (PGP) and their impact on the improving nutrient quality of the host plant are very poorly studied (8). However, we used two chickpea seed endophytes, *Enterobacter* sp. strains BHUJPCS-2 and BHUJPCS-8 (gene bank accession no. MN078044 and MN078047). In this study, our main objective is to (a) evaluate the impacts of the seed endophytes on the nutritional quality of the chickpea (edible part), (b) yield chickpeas after the application of these endophytes, and (c) grow plants under endophyte applications. We screened the nutritional values of different parts of chickpea plants, i.e., seeds, foliage, and pericarp (Figure 1). In this experiment, we have also compared the nutrient values of the seed endophyte-treated and non-treated plants. These experiments were conducted in control conditions by using clay pots to evaluate the effects of chickpea seed endophytes on host plants’ nutrient status.

## 2 Materials and methods

### 2.1 Isolation of endophytic microbes

Chickpea (*Cicer arietinum* L.) seed endophytes *Enterobacter* sp. BHUJPCS-2 (accession no MN078044) and *Enterobacter* sp. BHUJPCS-8 (accession no MN078047) were isolated from the chickpea seeds (Variety P-362) on the nutrient agar medium, according to Mukherjee et al. (17). These seed endophytes were screened on the basis of their PGP biochemical activities and PGP test in *in vitro* conditions (plant hormone production such as indole 3-acetic acid (IAA), mineral solubilisation, siderophore, hydrogen cyanide (HCN), protease productions, and showing antagonistic effect against *Fusarium* sp.). Seed endophytes *Enterobacter* sp. (BHUJPCS-2) and *Enterobacter* sp. (BHUJPCS-8) strains were identified through the 16 s rDNA amplification process using the colony polymerase chain reaction (PCR) method (17).

### 2.2 Endophytic inoculum preparation for seed treatment and sowing

Chickpea seeds (Variety - P-362) were used for the experiment. The seeds were washed by running tap water to remove the dust particles. Surface sterilisation of chickpea seeds was conducted by using 0.1% mercury chloride and ethanol, as described by Mukherjee et al. (10). The sterilised chickpea seeds were put on a sterilised Petri plate with sterilised double-distilled water (DDW) soaked in sterilised cotton and placed in the BOD incubator for 2–3 days at 25°C. For microbial cell preparation, strains BHUJPCS-2 and BHUJPCS-8 were inoculated in nutrient broth (NB) and incubated at 27°C ± 2°C in a shaker BOD incubator at 120 rpm for 72 h. Seed endophytes were collected in pellets by centrifugation (10,000 rpm for 5–10 min at 4°C). The suspended pellets were washed with sterilised DDW three to four times and maintained at a final cell concentration of 10^9^ mL^−1^ by measuring the absorbance of the bacterial suspension in a spectrophotometer. After maintaining the microbial cell concentration, the germinated seed and microbial cell of treatments T1 (*Enterobacter* sp. BHUJPCS-2) and T2 (*Enterobacter* sp. BHUJPCS-8) are mixed with the help of 1% carboxy methyl cellulose (CMC) (18) and C-(control) treatment without microbial cell. For seed sowing, we have used microbial cell-inoculated, healthy 15 germinated seeds in a pot. For this experiment, we have used 8 kg agricultural soil containing clay pots in triplicate.

### 2.3 Plant growth-promoting traits under clay pot condition

After observing the plant growth, three chickpea plants were randomly selected and uprooted from each clay pot after 110 days of planting. Plants and their roots were washed under tap water to remove soil and dust particles. The washed plants were air dried and then put into the oven at 50–55°C by wrapping the plant with blotting papers. Plants’ growth parameters (shoot and root length, fresh weight, and dry weight) were measured after 110 days of plant germination from each replication. Chickpea seed, pericarp, and foliage production were also observed and recorded.

### 2.4 Plant sample preparation

To prepare the sample, we collected the mature chickpea foliage, seed, and pericarp of each treatment from all the pots. The samples were mixed gently and dried at 50–55°C by wrapping the plant with a blotting sheet. After oven drying, the dried samples were ground thoroughly to make fine powder, and the powdered form of the sample was kept in a freezer (4°C) for further analysis.

### 2.5 Essential nutrient analysis of chickpea plant after endophytic treatment

Approximately 200 mg of dried powder of plant samples were used to analyse the nutrient content of the chickpea plant. The powdered plant samples were mixed in 5 mL of AR-graded sulphuric acid (concentrated H₂SO₄) in the conical flask. The flask was put in the shaker for mixing properly and placed at the normal room temperature for approximately 30 min. Then the sample containing the flask was boiled very gently for 30 min by adding 1 mL of perchloric acid (4% v/v). Then, the mixture was heated till it became transparent, and the samples were kept at room temperature for further use. This clear mixture of samples was further used for the analysis of phosphorus (P) by the colorimetric method (19). We have also analysed calcium (Ca), potassium (K), and sodium (Na) by inductively coupled plasma (ICP, PerkinElmer). In this study, we have used a 2:1 ratio of nitric acid (65%) and perchloric acid (70%) for acid digestion of plant samples for ICP (20). Total nitrogen (N) and organic matter (OM) were measured (21, 22). Proteins were estimated using the Bradford method, and carbohydrates in the plant sample were measured using the anthrone reagent (23, 24).

### 2.6 Detection of total phenol content (TPC) and total flavonoid content (TFC) of chickpea plants after endophytic bacterial inoculation

The TPC of the chickpea plant samples was measured by using the Zheng and Shetty (25) methods. From each sample (pericarp, foliage, and seed), 0.1 g was taken from all replications and incubated with 5 mL of 95% ethanol at 0°C for 48 h. These plant samples were then homogenised and centrifuged at 15000 rpm for 15 min in a cooling centrifuge, specifically the Eppendorf-5430R. One millilitre (1 mL) of the supernatant sample was collected and to that, 1 mL of 95% ethanol was added along with 5 mL of sterilised DDW, and then 0.5 mL of 50% Folin–Ciocalteu reagent was added. This whole sample–reagent mixture was mixed properly. After 10 min of incubation, 1 mL of sodium carbonate (5% v/w) was added to the mixture, and the whole mixture was incubated at room temperature for 1 h. The absorbance of the colour change was recorded at 725 nm (Genetix, Nabi Spectrophotometer, NB- 1-181007, Korea).

To detect flavonoids in plants, the sample was prepared by mixing 2 g of oven-dried powders of plant samples (pericarp, foliage, and seeds) separately in 10 mL of 50% methanol and incubating the mixed sample overnight at room temperature. The incubated samples were filtered with the help of sterilised Whatman’s No. 1 filter paper. The filtered samples were fractionated with C_4_H_8_O_2_ (ethyl acetate). After fractionation, the remaining fractionated residue was re-fractionated using an equal volume of ethyl acetate. Then, the fractionated samples were evaporated to dryness. Then, the dried samples were dissolved in 2 mL of HPLC-grade (Shimadzu LC-10A, Japan) methanol and used for further analysis (8). TFC was quantified in the plant samples by using 0.5 mL of sample extract. For the extract, 4 mL of sterilised DDW and 0.3 mL of 50% of NaNO_2_ solution were added and mixed. After 10 min of incubation, 0.3 mL of AlCl_3_ (10%) solution was added to all the samples and mixed gently. After that, the samples were incubated for another 10 min. Then, 2 mL of 1 M NaOH was added to that sample and the final volume was 10 mL with 95% ethanol. Then, the solution was mixed properly and the absorbance was measured at 510 nm (26, 27). High-performance liquid chromatography (HPLC) of plant samples (seed) was performed to detect the phenolic compounds (28).

### 2.7 Statistical analysis

All experiments were conducted in triplicates, and the results were prepared as the mean ± standard deviation (SD) of the different independent replicates. In this study, we have used Duncan’s multiple range test (DMRT) for the statistical analysis in SPSS version 20.

## 3 Results

### 3.1 Chickpea plant growth, biomass, and yield after endophyte treatment

**Figure 2:**
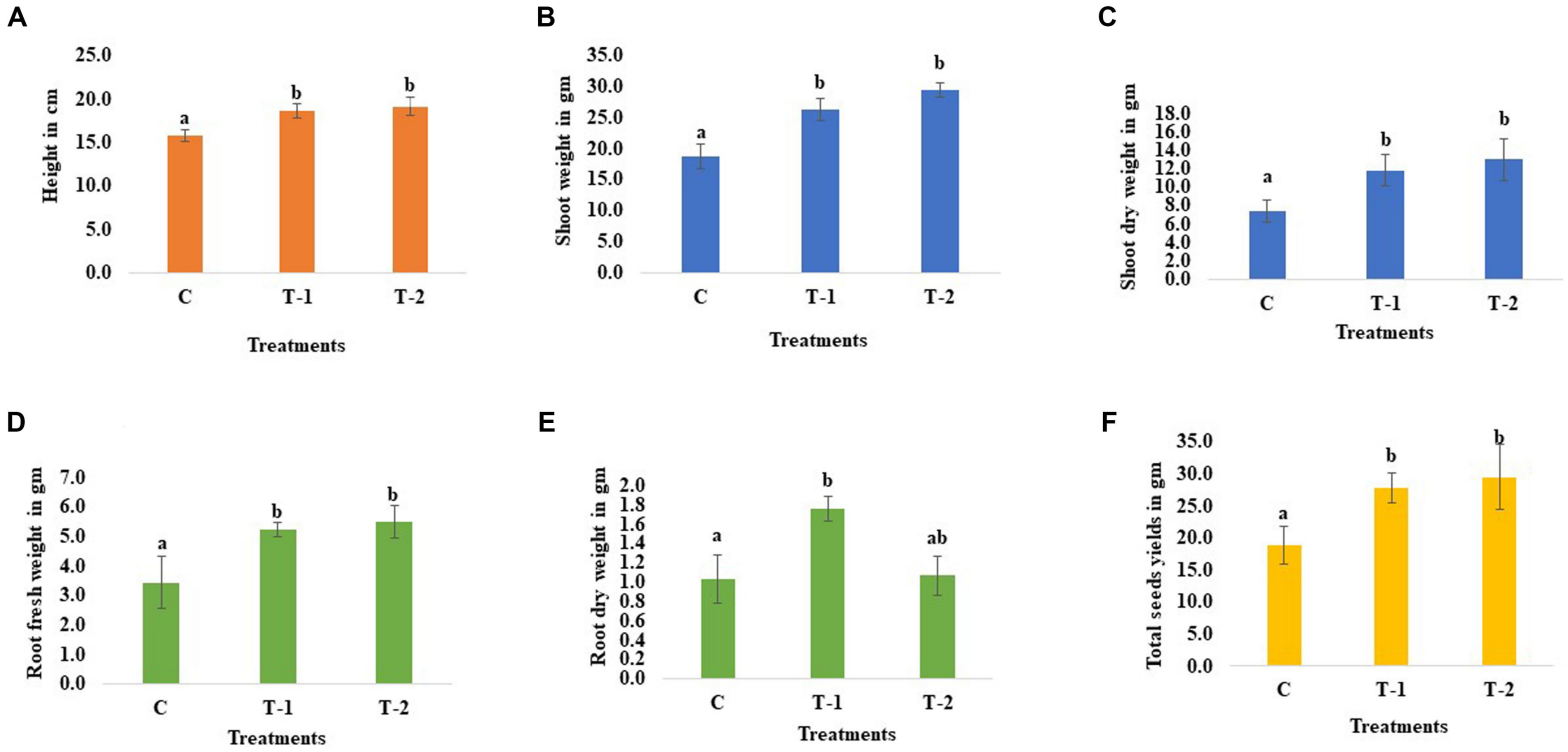
Effect of chickpea seed endophytes *Enterobacter* sp. BHUJPCS-2 and *Enterobacter* sp. BHUJPCS-8 on the **(A)** plant height, **(B,C)** shoot fresh and dry weight, **(D,E)** root fresh and dry weight, and **(F)** grain yields. *The data values are the mean ± SD; mean values in each column with the same superscript(s) do not differ significantly by Duncan’s multiple *post-hoc* test (*p* = 0.05). Here, cm = centimetre, gm = gram, control (without microbial treatment) or C, *Enterobacter* sp. BHUJPCS-2 (T-1) and *Enterobacter* sp. BHUJPCS-8 (T-2).

The microbes were isolated from the chickpea seeds (P-362), and the 16 s rDNA sequencing reveals that the isolated bacteria belong to *Enterobacter* sp. BHUJPCS-2 (accession no MN078044) and *Enterobacter* sp. BHUJPCS-8 (accession no MN078047), which were previously discussed in our research article (17). The height of the chickpea plant in the clay pot experiment was increased by 0.18- and 0.21-folds in endophyte-treated plants than the untreated control plants. After harvesting the plant from the pot, we observed that the shoot fresh weight was increased by 0.41- and 0.57-folds, the shoot dry weight was increased by 0.59- and 0.75-folds, root fresh weight was increased by 0.54- and 0.61-folds, and the root dry weight was increased by 0.70- and 0.02-folds in the endophyte-treated chickpea plant compared to the control plants. The grain yield was also observed, with a 0.47- and 0.56-fold increase in endophyte-treated plant compared to the control plant (Figure 2).

### 3.2 Effect of endophytic inoculum on plant nutrients and other important beneficial biochemical components

Important nutrients such as Na, Ca, N, P, K, protein, carbohydrate, flavonoid, and phenolic components in chickpea seed, pericarp, and foliage were checked both in the endophyte-treated and endophyte-untreated plants. In this study, we observed that nutrient contents were improved in the treated plants. The influence of the endophytic microbes on protein, carbohydrates, total phenolics, and flavonoids was also observed.

### 3.3 Effect of endophytes on some macronutrients (N, P, Ka, ca, and Na) of chickpea edible part

Total phosphate (P) contained in pericarp (0.27- and 0.32-folds), seed (0.03- and 0.12-folds), and foliage (0.46- and 0.12-folds) was increased in the endophytes *Enterobacter* sp. BHUJPCS-2- and *Enterobacter* sp. BHUJPCS-8-treated chickpea plants, respectively, than control plants. In this study, maximum P content was observed in the chickpea foliage part of the plant, followed by the pericarp and seeds. A similar type of result was observed in the other two nutrients, K and N. K content was improved in pericarp (0.10- and 0.16-folds), foliage (0.054- and 0.11-folds), and seed (0.041- and 0.25-folds) in the BHUJPCS-2- and BHUJPCS-8-treated plant compared to the control. N content was also observed and found that in the pericarp, the N was 0.923- and 1.79-fold increase, where 0.27- and 0.44-fold increase, 0.36- and 0.69-fold increase in seed and foliage, respectively, in the BHUJPCS-2- and BHUJPCS-8-treated plants than control plants (Figure 2).

**Figure 3:**
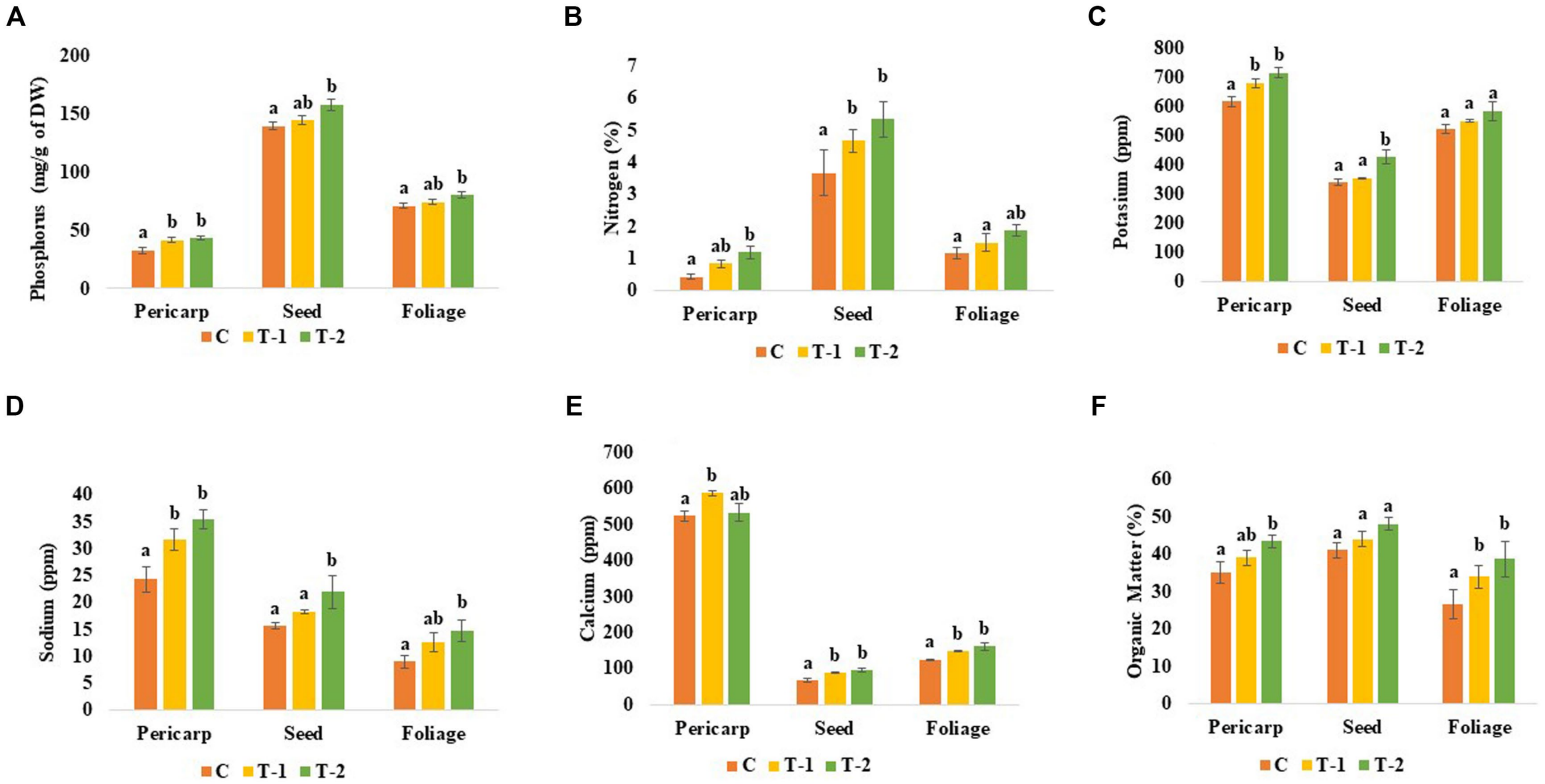
Effect of chickpea seed endophytes *Enterobacter* sp. BHUJPCS-2 and *Enterobacter* sp. BHUJPCS-8 on the **(A)** phosphorus, **(B)** nitrogen, **(C)** potassium, **(D)** sodium, **(E)** calcium, and **(F)** organic matter (OM) content of different edible part such as pericarp, seed, and foliage of chickpea plants. *The data values are the mean ± SD; mean values in each column with the same superscript(s) do not differ significantly by Duncan’s multiple *post-hoc* test (p = 0.05). Here, mg = milligram, g = gram, % = percent, ppm = parts per million, control (without microbial treatment) or C, *Enterobacter* sp. BHUJPCS-2 (T-1) and *Enterobacter* sp. BHUJPCS-8 (T-2).

Other important essential nutrients, such as Na and Ca, are also measured in the major edible parts of the chickpea, such as pericarp, seed, and foliage. We observed that 0.30- and 0.45-fold Na was increased in the pericarp, 0.17- and 0.40-fold increase in seed, and 0.40- and 0.62-fold increase in the foliage part of the endophytes BHUJPCS-2- and BHUJPCS-8-treated plants than control plants. The Ca contain was increased 0.10 and 0.16 fold in pericarp, 0.31 and 0.41 fold in seed and 0.19 and 0.29 fold in the foliage part in strains BHUJPCS-2 and BHUJPCS-8 treated plants, respectively as compared to the control plants (Figure 3).

### 3.4 Effect of endophytes on organic matter and phenols contained in chickpea plant

Total phenol content increased 0.25- and 0.07-folds in pericarp, 0.33- and 0.55-folds in seed, and 0.30- and 0.47-folds in the foliage of BHUJPCS-2 and BHUJPCS-8 endophyte-treated plants compared to the control plants. OM of the pericarp was increased 0.11- and 0.12-folds, 0.07- and 0.17-folds in seed, and 0.27- and 0.45-folds in the foliage of BHUJPCS-2 and BHUJPCS-8-treated chickpea plants compared to the control-untreated plants (Figure 3).

### 3.5 Effect of endophytes on protein, carbohydrate, and flavonoid content in chickpeas

**Figure 4:**
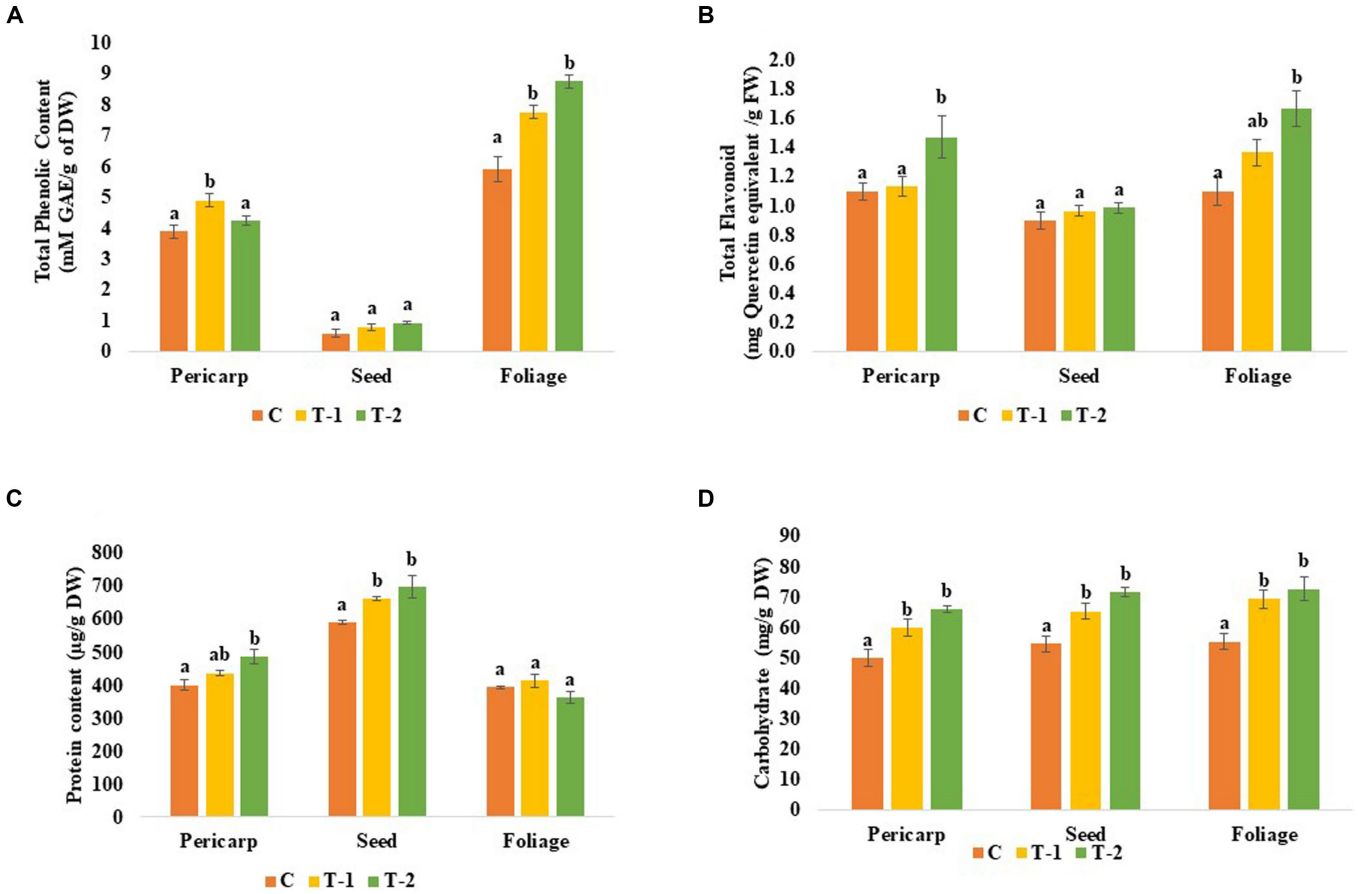
Effect of chickpea seed endophytes *Enterobacter* sp. BHUJPCS-2 and *Enterobacter* sp. BHUJPCS-8 on the **(A)** phenolic content, **(B)** flavonoid content, **(C)** protein content, and **(D)** carbohydrate content in the pericarp, seed, and foliage of chickpea plants. ^*^The data values are the mean ± SD; mean values in each column with the same superscript(s) do not differ significantly using Duncan’s multiple *post-hoc* test (*p* = 0.05). Here, control (without microbial treatment) or C, *Enterobacter* sp. BHUJPCS-2 (T-1) and *Enterobacter* sp. BHUJPCS-8 (T-2).

Microbe-treated plants contain higher amounts of protein and carbohydrates in the edible part of the plant. Protein content of the pericarp was increased by 0.09- and 0.21-folds where 0.11-, 0.18-, and 0.048-folds increased, respectively, in the seed and foliage parts of BHUJPCS-2 and BHUJPCS-8-treated plants compared to the control untreated plants. A similar type result was also observed in the carbohydrate. In this study, we found a 0.2- and 0.32 = fold increase in the pericarp, 0.19- and 0.31-fold increase in seed, and 0.25- and 0.31-fold increase in the foliage part of the BHUJPCS-2- and BHUJPCS-8-treated plant, respectively, compared to the control plant. In the flavonoid content, we measured 0.02- and 0.27-fold increase in pericarp, 0.06- and 0.08-folds in the seed, and 0.23- and 0.45-fold increase in the foliage part in endophyte-treated plants compared to the control plants (Figures 3, 4).

### 3.6 HPLC analysis of phenolic compounds in chickpea seeds

Different kinds of phenolic components were estimated through the HPLC in the chickpea seeds of endophyte-treated and endophyte-untreated chickpea plants. We mainly focussed on shikimic acid, gallic acid, syringic acid, ferulic acid, p-coumaric acid, and cinnamic acid. In HPLC, we found that only the p-coumaric acid concentration was higher in the control seeds than the endophytic-treated seeds, but other components were higher in the endophyte-treated seeds. In this study, shikimic acid was increased by 0.24- and 0.30-folds, gallic acid increased by 0.27- and 0.41-folds, ferulic acid increased by 0.25- and 0.37-folds, cinnamic acid increased by 0.28- and 0.42-folds, syringic acid increased by 0.37-fold (only in BHUJPCS-2-treated plants), and p-Coumaric increased by 0.07-fold (only in BHUJPCS-8-treated plants) in BHUJPCS-2 and BHUJPCS-8 endophytic-treated plants than the control plant (Table. 1).

**Table 1:** Detection of shikimic acid, gallic acid, ferulic acid, syringic acid, p-coumaric acid, and cinnamic acid of different parts (seed, foliage, and pericarp) of chickpea plant which were raised after endophytic microbial treatments.

Chickpea Seed (μg/ml)						
Treatment	Shikimic acid	Gallic acid	Ferulic acid	Syringic acid	p-Coumaric acid	Cinnamic acid
Control	28.8 ± 1.07^a^	3.3 ± 0.30^a^	0.11 ± 0.03^a^	1.6 ± 0.05^a^	1.4 ± 0.23^a^	0.16 ± 0.02^a^
T-1	33.8 ± 3.23^b^	5.1 ± 0.61^b^	0.16 ± 0.05^ab^	2.0 ± 0.20^b^	0.9 ± 0.10^b^	0.19 ± 0.01^b^
T-2	36.9 ± 1.02^b^	5.4 ± 0.43^b^	0.23 ± 0.01^b^	1.2 ± 0.02^b^	1.7 ± 0.30^b^	0.21 ± 0.03^b^

Chickpea Pericarp (μg/ml)						
Treatment	Shikimic acid	Gallic acid	Ferulic acid	Syringic acid	p-Coumaric acid	Cinnamic acid
Control	33.43 ± 4.22^a^	2.63 ± 0.25^a^	0.16 ± 0.05^a^	1.80 ± 0.11^ab^	1.71 ± 0.27^a^	0.16 ± 0.02^a^
T-1	34.13 ± 3.49^a^	5.13 ± 0.33^b^	0.17 ± 0.02^a^	2.00 ± 0.04^b^	1.32 ± 0.19^a^	0.12 ± 0.06^a^
T-2	38.65 ± 0.90^a^	4.71 ± 0.61^b^	0.23 ± 0.01^b^	1.37 ± 0.05^a^	2.03 ± 0.15^a^	0.21 ± 0.03^b^

Chickpea Foliage (μg/ml)						
Treatment	Shikimic acid	Gallic acid	Ferulic acid	Syringic acid	p-Coumaric acid	Cinnamic acid
Control	29.43 ± 3.06^a^	3.3 ± 0.36^a^	0.40 ± 0.02^a^	2.2 ± 0.60^a^	1.51 ± 0.04^a^	0.17 ± 0.04^a^
T-1	30.13 ± 1.97^ab^	5.8 ± 0.02^b^	0.59 ± 0.03^b^	2.4 ± 0.09^a^	1.99 ± 0.09^ab^	0.22 ± 0.04^ab^
T-2	35.35 ± 3.07^b^	5.3 ± 0.54^b^	0.37 ± 0.01^a^	1.9 ± 0.09^a^	2.57 ± 0.05^b^	0.25 ± 0.02^b^

^*^The data values are the mean ± SD; mean values in each column with the same superscript(s) do not differ significantly using Duncan’s multiple post-hoc test (*p* ≤ 0.05). Here, Enterobacter sp. BHUJPCS-2 (T-1) and Enterobacter sp. BHUJPCS-8 (T-2).

## 4 Discussion

Chickpeas are one of the healthiest food commodities around the world, but to date, very little is known about chickpeas’ health benefits compared to other leguminous plants (29). Some earlier research studies suggested that chickpea consumption improves health by reducing the risk of different diseases (49), and it can also reduce the level of total cholesterol in the serum (30) and the risk of coronary heart disease (CHD) (31). Scientists are trying to improve the nutritional values of food products by improving plant breeding programmes, improving biotechnological interventions for different food products. Although the rhizosphere beneficial microbe-induced plant health and defence mechanisms are very well studied (47), the studies linked to seed endophytic bacteria-induced growth and nutritional value in different parts of crops are still lacking. Therefore, in our present study, we checked the effects of the seed endophytic microbe *Enterobacter* sp. (BHUJPCS-2 and BHUJPCS-8) on enhancing the nutritional value of the edible parts of chickpeas.

Chickpea seeds are mostly consumed by humans, whilst the foliage and pericarp serve as fodder in many countries around the world. It has been shown that chickpeas are full of nutrients such as N, P, and K, which are the most important inorganic minerals that are essential for growth (14, 48). These are also the important constituents of several important components (proteins, enzymes, hormones, and amino acids) and genetic materials (32, 33). Host plant-competent endophytes play an important role in providing available forms of nutrients to the host plant (6, 11, 50). In our experiment, we noticed a direct correlation between increased dry weight and the nutrient content (proteins and carbohydrates) of the seeds, and the microbial treatments increased the total P and N content. As our endophytic microbes are able to solubilise the minerals and improve the nutrient uptake of the plant, it may influence the improvement of the nutrient condition in the edible parts of the plant.

We used endophytic *Enterobacter* isolated from chickpea seeds, these microbes are normally found in plants, soil, water, and in the gastrointestinal tracts of animals. *Enterobacter* sp. have been found to promote plant growth, and they have been studied for their potential as biofertilisers (34, 51). *Enterobacter* strains with PGP properties are thought to work through a variety of mechanisms (34, 52). The most important mechanism is the synthesis of PGP- hormones such as indole-3 acetic acid (IAA), which can stimulate root growth and enhance nutrient uptake by the plant (16). Another mechanism is through the production of enzymes such as phosphate, K, and zinc solubilizing enzymes, which can release nutrients from organic sources in the soil and make them available to the host plants (12, 17, 35). In addition to producing PGP substances, *Enterobacter* also acts as a biocontrol agent against plant pathogens (51). Some strains of *Enterobacter* have been shown to produce antibiotics and siderophores that can inhibit the growth of plant pathogens (36). In our pot experiments, we observed that an increase in plant growth, OM, and mineral uptake was correlated with the carbohydrate and protein content of the endophyte-treated plant. Additionally, the total protein of seeds was directly correlated with the flavonoid and OM of the pericarp and seed, respectively. These positive correlations could be directly attributed to the improved uptake of different important nutrients and minerals, including N, P, K, Ca, and Na, by host chickpea plants in endophyte treatments compared to the control plants. Interestingly, the host chickpea plants treated by the endophytes showed a maximum increase in the mineral and nutrient contents, showing the advantage of endophyte treatment. This type of study was conducted with white beans under stress conditions and observed similar results (37). Increased N content was also reported in the chickpea plants treated with *Microbispora* sp. (strain CP56), *Actinomadura* sp. (strain CP84B), and *Streptomyces* spp. (strain CP200B and strain CP21A) (38). Humans and other animals consume most of their nutrients from different food products. So, having a lot of nutrients in different plant parts is important because they have an impact on our daily diets (52). In our study, an increase in the total protein, carbohydrate, phenolics, and flavonoids in the microbial-treated chickpea plant (seed, pericarp, and foliage) compared to control plants is the real indication of improved nutrition in the endophyte-treated plants.

Higher phenolic accumulation in endophyte-treated host plants is highly noteworthy from a beneficial perspective since phenols are the essential tool for plant defence against different invading pathogens and are directly related to the free radical scavenging property (53). Similarly, polyphenols are also directly involved in the signal transduction and perception processes of different pathways, and they also change the cellular redox potential conditions (39). Higher accumulation of the phenolics was studied in the fenugreek plant when the host plants were treated with endophyte seed microbes, *Achromobacter* sp. (40). Shikimic acid is also a very important component, as it acts as the precursor of most phenolic compounds (54). It is very interesting to note that the higher shikimic acid accumulation resulted from the enhanced phenylpropanoid activities. It is directly involved in higher phenolic synthesis. Gallic acid, ferulic acid, cinnamic acid, and syringic acid are all phenolic compounds found in plants. These compounds play important roles in the metabolism and physiology of plants and are also known for their various health benefits for humans (41). Gallic acid is a potent antioxidant found in many plant-based foods, such as grapes, blueberries, and tea. It is also found in some medicinal herbs, such as *Terminalia chebula*, which is used in Ayurvedic medicine (42). Gallic acid has been shown to have anti-inflammatory/cancer/microbial properties (55). It is also known to help regulate blood sugar levels and improve cardiovascular health. Ferulic acid is an important component of the plant cell wall and is found in many grains, such as wheat, rice, and corn, as well as in fruits and vegetables. It is known to have antioxidant and anti-inflammatory properties and has been shown to improve skin health and reduce the risks of certain chronic diseases, such as diabetes and heart-related disease (43). Cinnamic acid is found in many plants, including cinnamon, and is known to have anti-inflammatory as well as anti-microbial properties. It has also been shown to help regulate diabetes (blood sugar levels) and improve heart health (44). Syringic acid is found in many fruits and vegetables, including grapes, strawberries, and sweet potatoes. It is known to have antioxidant properties and has been shown to have anti-inflammatory and anti-cancer effects. It may also have potential therapeutic applications for diabetes and cardiovascular disease (45). These types of flavonoids are also known to work as potential antioxidants and also improve the antioxidant properties of food by restricting the activity of other oxidases (46, 56). Due to erythrocyte membrane malfunction, various types of flavonoids in the diet diminish lipid peroxidation and the permeability of K (57). The increasing use of cereals as livestock feed creates a very competitive situation with human feed. Humans and livestock are growing rapidly around the world to meet the basic requirements of a proper diet, both for humans and livestock. The demand for food and fodder has continuously increased. Thus, additional resources of proper nutrients, such as chickpea seed and straw, have been accepted as feed for humans and livestock. Therefore, the use of chickpea seed, pericarp, and foliage is considered the best alternative food to overcome this major problem because of their high nutritional value. The current small experimental study thus suggests that the application of seed endophytes improves the nutrient content in the different parts of plants and also improves yield.

## 5 Conclusion

One of the significant global health problems affecting billions of people worldwide is nutrient deficiency in their diets. Due to low concentrations and poor bioavailability of vital micronutrients contained in their frequently consumed foods, the majority of populations in underdeveloped countries are deficient in one or more critical vitamins and minerals. Strategies to provide nutrient-dense diets and enhance nutrient concentrations and bioavailability are needed to reverse the epidemic of micronutrient malnutrition in emerging nations. Hence, chickpeas are one of the potential crops in terms of providing a complete nutritional remedy for micronutrient deficiencies in poor nations. In this study, we provide evidence and demonstrate that chickpea seed endophytes have a high number of plant growth-promoting traits that can be potentially used to enhance the quality of chickpeas. In this context, the endophytes used in this study, upon re-introduction with the seeds, enhanced plant growth, yield, and nutritional values. Looking ahead, further research into harnessing the potential of chickpea seed endophytes holds promise for tackling global malnutrition. Future studies could delve deeper into understanding the mechanisms behind the enhanced nutritional qualities observed and explore broader applications of these findings in sustainable agriculture. Additionally, field trials and large-scale implementation of bio-inoculants derived from endophytes could offer practical solutions for improving food security and public health in regions prone to nutrient deficiencies. Therefore, these two bacterial isolates have some important characteristics for developing effective bio-inoculants that can be tested later for their activity in field conditions.

## Data availability statement

The datasets presented in this study can be found in online repositories. The names of the repository/repositories and accession number(s) can be found in the article/supplementary material.

## Author contributions

AM: Conceptualization, Methodology, Writing – review & editing, Data curation, Formal analysis, Software, Validation, Visualization, Writing – original draft. AG: Data curation, Formal analysis, Writing – review & editing. GC: Data curation, Formal analysis, Writing – review & editing. SS: Data curation, Formal analysis, Software, Writing – review & editing. AS: Formal analysis, Writing – review & editing. SA: Writing – review & editing. JV: Conceptualization, Funding acquisition, Investigation, Methodology, Project administration, Resources, Supervision, Writing – review & editing.
